# A Case Series of Oral Cancer: An Enigma to Diagnosing Clinicians

**DOI:** 10.7759/cureus.112662

**Published:** 2026-07-14

**Authors:** Ramachandra Reddy Gowda Venkatesha, Karthik Rajaram Mohan, Saramma Mathew Fenn, Reethika Rathan, Kumar Appusamy

**Affiliations:** 1 Oral Medicine and Radiology, Vinayaka Mission's Sankarachariyar Dental College, Vinayaka Mission's Research Foundation (Deemed to Be University), Salem, IND

**Keywords:** chronic ulcer, floor of mouth, oral cancers, retromolar pad area, scc (squamous cell carcinoma) buccal mucosa

## Abstract

Oral cancer is one of the most common health hazards encountered among the South Asian population. Oral cancer ranks sixth among other cancers worldwide. The risk factors for oral cancer are usually multifactorial, and the distribution of oral cancer varies according to ethnic origin. Risk factors for oral cancer include tobacco use (smoking and chewing), excessive alcohol consumption, betel nut chewing, human papillomavirus (HPV) infection, and a diet low in fruits and vegetables. Oral cancer significantly impacts both the quality of life and the survival of affected individuals. Oral cancer refers to cancers that develop in the tissues of the mouth or throat, including the lips, tongue, gums, floor of the mouth, and roof of the mouth. It can manifest as a sore, lump, or ulcer in the mouth that does not heal, difficulty or pain with swallowing, persistent mouth pain, or changes in voice. Early detection and treatment of oral cancers are crucial for improving outcomes in oral cancer patients. Unfortunately, many cases are diagnosed at advanced stages, which reduces the chances of successful treatment and increases morbidity and mortality rates. Dentists and healthcare providers play a critical role in the early detection of oral cancer through regular screenings, especially in high-risk individuals. Early detected stage I cancer has a better prognosis than newly diagnosed stage III or stage IV cancer, which usually affects quality of life, resulting in poor survival and mortality. This article provides a comprehensive overview of the clinical features of oral cancer in different regions of the oral cavity and the importance of early diagnosis of this neglected entity.

## Introduction

The Greek word "Karkinos" (kar-hard, meaning crab) refers to malignant tumors that clinically resemble the hard, swollen legs of a crab due to enlarged veins affecting the limb; the Latin word "crab" means creeping ulcer. This is where the word "cancer" originates. In his work "On Tumors Against Nature," the Greek physician, philosopher, and surgeon Galen of Pergamon classified abnormal and unnatural body masses in great detail. He explained that these masses were the result of an elevated concentration of black bile from the liver in the affected tissue. He proposed that neoplasms were caused by the accumulation of extra black bile in specific tissues, such as the tongue and lips. He coined the term "volume/swelling" (gos/goma) to characterize the tumors [1].

The final section of the Ebers Papyrus, "Treatise on Tumors," describes numerous cancerous lesions as "an eating ulcer on the gum," using the ancient Egyptian term "bnwt" [2]. In his 1903 work "Les tumeurs" (the tumors), Duplay and Cazin distinguished benign lesions from malignant ones by referring to cancer as “a mass constituted of newly developed tissue, tending to persist or increase.” He also unequivocally confirmed the link between tobacco use, malnutrition, and mouth cancer. Additionally, they discovered that "leukoplakia," which manifests as a solid, nonscrapable white patch on the oral mucosa near the commissural region, is a significant risk factor for cancer [3].

Sir William Temple, in 1960, described cancer as a corroding or sloughing gangrenous ulcer that corrodes or destroys adjacent structures. Oral cancer is characterized by an atypical mass of tissue whose growth surpasses that of the surrounding tissue and continues to grow excessively even after the stimuli triggering the change cease to exist. Metastasis, a major cause of death from oral cancer, occurs when the abnormal mass of tissue grows uncontrollably beyond its boundaries and invades the adjoining parts of the organ [4].

## Case presentation

Case 1

A 57-year-old male reported a chief complaint of soreness while eating spicy foods and sudden hoarseness of his voice for the past month. His medical history revealed that he accidentally avulsed his mandibular third molar while brushing with a neem stick a month ago. His medical history was non-contributory. The patient has been a chronic bidi smoker for approximately 17 years and smokes about three bidis per day, according to his history. An extraoral examination revealed a single palpable right submandibular lymph node approximately 1 cm in diameter, stony-hard in consistency, and fixed. His vitals are stable. An intraoral examination revealed an ulceroproliferative growth measuring approximately 7.5 cm x 6.5 cm in the right retromolar area and posterolateral border of the tongue (Figure 1).

**Figure 1:**
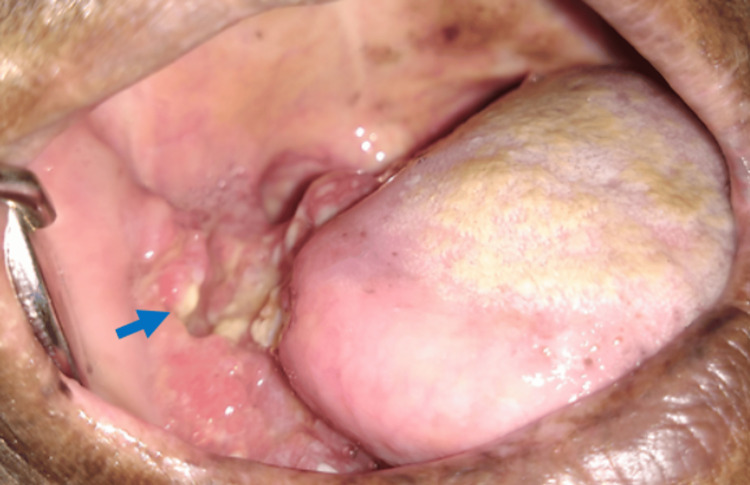
An intraoral examination revealed an ulceroproliferative growth involving the right retromolar pad region and posterolateral border of the tongue (blue arrow).

His blood tests for white blood cell (WBC) count, differential count, erythrocyte sedimentation rate (ESR), bleeding time, and clotting time were within normal ranges. The orthopantomography revealed mandibular alveolar bone destruction with a pathological fracture (Figure 2).

**Figure 2:**
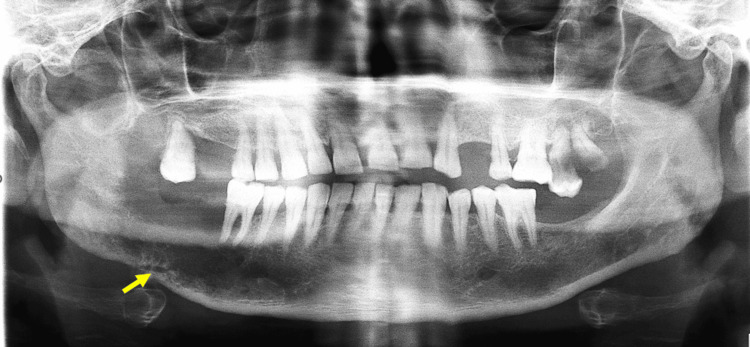
The orthopantomography image revealed mandibular alveolar bone erosion and pathological jaw fracture caused by oral cancer (yellow arrow).

The axial section computed tomography (CT) scan revealed destruction caused by erosion of the underlying mandibular alveolar bone due to the ulceroproliferative growth in the molar region (Figure 3).

**Figure 3:**
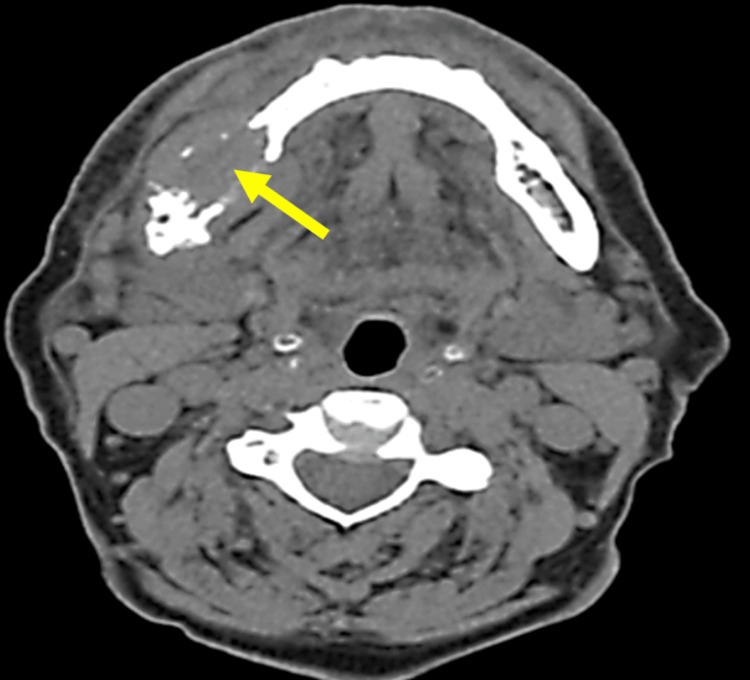
The axial section of the computed tomography image revealed the destruction of the mandibular alveolar bone in the right mandibular molar region (yellow arrow).

The sagittal section of the CT image revealed a floating-tooth appearance (Figure 4).

**Figure 4:**
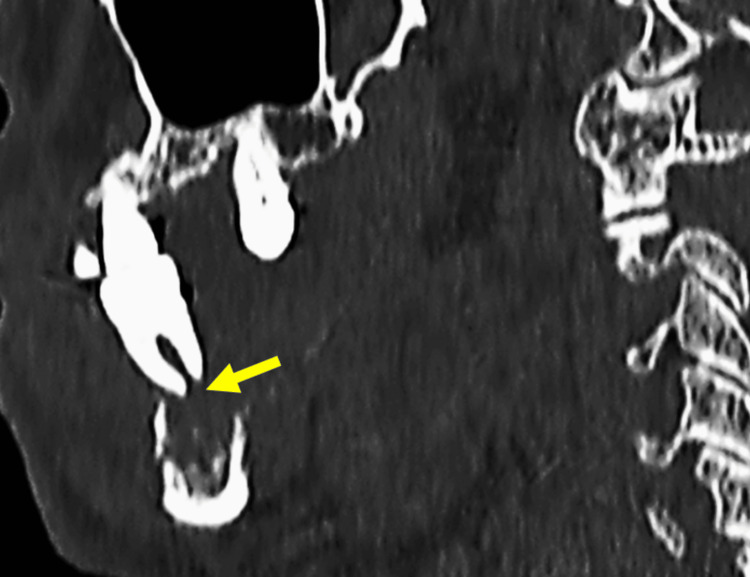
The sagittal section of the computed tomography image revealed a "floating-tooth" appearance (yellow arrow).

The staging of oral cancers in reported oral cancer cases was done based on the size of the tumor (T), lymph node (N), and metastases (M) using the TNM staging system developed by Kato et al. (2020) (Table 1) [5].

**Table 1:** TNM staging of oral cancer by Kato et al. (2020). The table was modified and created with permission from the authors [5].

American Joint Committee on Cancer Staging of Oral Cancer	
T–the size of the primary tumor	T1: size <2cm and depth of invasion (DOI) <5mm T2: size 2 cm and DOI 5-10 mm or size 2-4 cm and DOI <10mm T3: size >4 cm or any tumor >10 mm DOI
N–lymph node	No = no lymph node involved N1 = single ipsilateral lymph node <3 cm in size N2 N2a: single ipsilateral lymph node 3-6 cm in size. N2b: Multiple ipsilateral lymph nodes, all <6 cm in size. N2c: any bilateral or contralateral lymph nodes, all <6 cm in size. N3: any lymph node >6 cm in size. Clinical N0-N2: same as previous and extranodal extension (ENE)(-) N3 now with subcategories: N3a: previous N3 (size >6 cm) and ENE (-) N3b: any ENE(D), either clinical or radiographic Pathologic criteria for pathologic ENE (+) oral cavity: only macroscopic (>2 mm) oropharynx: micro (2 mm) or macroscopic (>2 mm) N1-N2: same as previous and ENE (-) with exception: N2a includes lymphnode <3 cm, ENE (+) LN N3 now with subcategories: N3a is previous N3 (size >6 cm) and ENE (-); N3b: >3 cm and ENE (+) LN or >1 ENE (+) LNs
M–metastases	MX = metastases cannot be assessed M0 means the cancer has not spread to other parts of the body M1 means cancer has spread to other parts of the body such as the lungs

The TNM staging assessment revealed a T3N1Mx status. The patient was referred to the cancer institute for further management.

Case 2

A 52-year-old male reported a chief complaint of soreness in his tongue for the past two months. History of presenting illness: the ulcer started at a small size and has progressed to the present size. The patient has been a chronic bidi smoker for about 12 years and smokes about seven bidis per day. The intraoral clinical examination revealed an ulceroproliferative growth measuring approximately 2 cm x 1.5 cm in diameter with everted margins; the floor shows necrotic slough (Figure 5).

**Figure 5:**
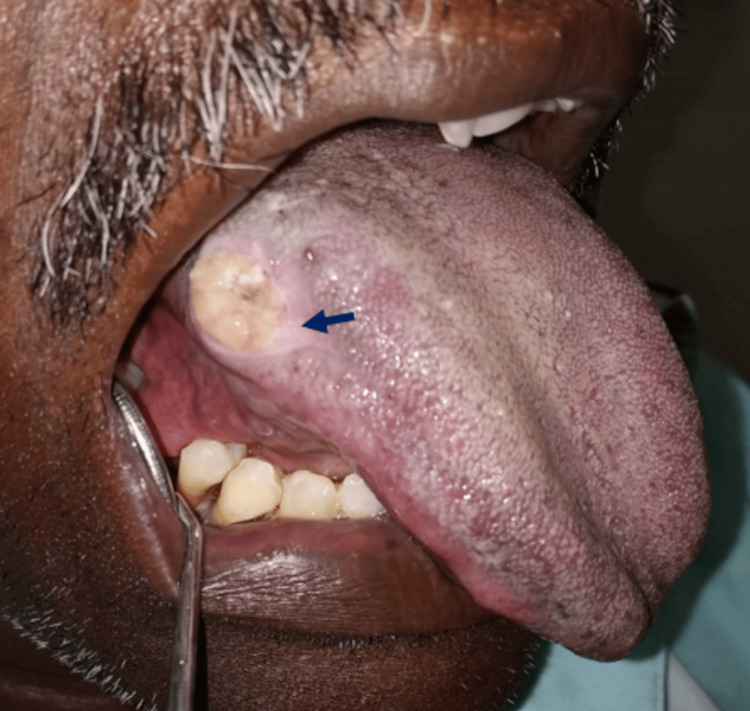
An intraoral clinical examination revealed an ulceroproliferative growth measuring 1 cm x 1.5 cm in diameter involving the right posterolateral border of the tongue (blue arrow).

All the inspectory findings regarding the number, site, size, shape, surface, and extent were confirmed on palpation; it was non-tender and base-indurated. Blood investigations were within biological ranges. The size of the lesion and clinical findings led to the TNM staging assessment of T2N0Mx. We planned a partial glossectomy and adjuvant chemoradiotherapy for this patient. We referred the patient to a cancer institute for further management.

Case 3

A 62-year-old male reported a chief complaint of soreness while eating food for the past two months. History revealed that the patient had been a chronic bidi smoker for the past 18 years. He smokes about 12 bidis per day. An intraoral examination revealed an irregular ulceroproliferative growth measuring approximately 2 cm x 1.5 cm on the right buccal mucosa in relation to the right mandibular second and third molar regions (Figure 6).

**Figure 6:**
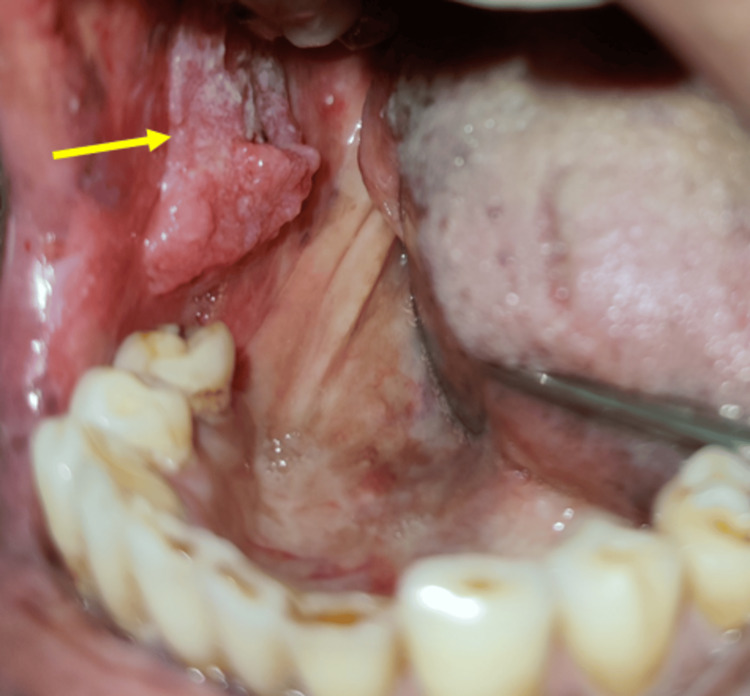
An intraoral examination revealed an ulceroproliferative growth on the right buccal mucosa (yellow arrow).

All the inspectory findings, including the number, site, size, shape, surface, and extent, were confirmed through palpation, and the growth was indurated. Blood tests revealed he has diabetes, with a postprandial blood glucose level of 247 mg/dL. We assessed the TNM staging as T2N0Mx. The treatment plan involved surgery, followed by adjuvant chemoradiotherapy. The patient was referred to a cancer institute for further management.

Case 4

A 48-year-old male reported a chief complaint of sudden spacing between his lower front teeth. History revealed that he noticed a small growth measuring approximately 4.5 cm x 1.5 cm, which started as a small one two days ago and has suddenly progressed to the present size within a very short time. The patient has been a chronic cigarette smoker for about seven years. He smokes about seven cigarettes per day. An extraoral examination revealed a single palpable submental lymph node approximately 1 cm in diameter, which is stony-hard in consistency, non-tender, and fixed. An intraoral examination revealed a growth on the gingiva, causing the pathological migration or spacing of his mandibular anterior teeth and also the stretching of the marginal and attached gingiva in the mandibular canine and first premolar regions (Figure 7).

**Figure 7:**
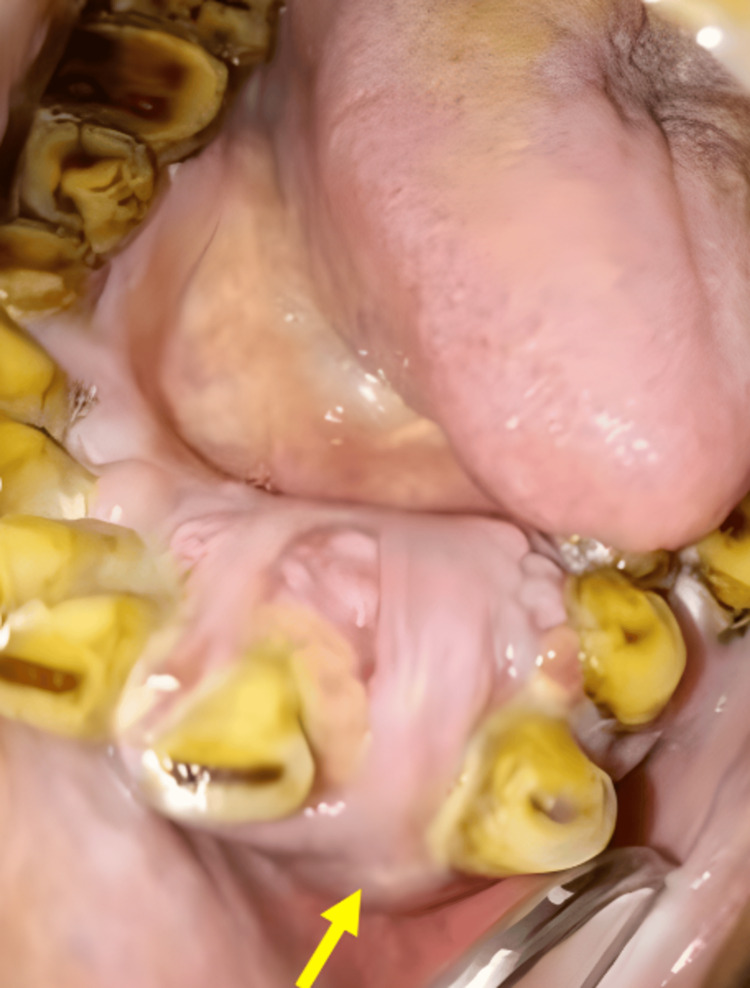
An intraoral examination revealed a growth on the gingiva, stretching the marginal, attached gingiva and also causing the sudden drifting or pathological migration of the mandibular anterior teeth.

Blood investigations were within biological ranges. In this case, we assessed the TNM staging as T3N1Mx. In this case, the planned treatment involved hemimandibulectomy, followed by adjuvant chemoradiotherapy. We referred the patient to a cancer institute for further management.

Clinical significance

Oral cancer is clinically significant due to its potential for serious morbidity and mortality, the importance of early detection and prevention, diagnostic challenges, the range of treatment modalities available, functional and aesthetic impacts, and its psychosocial effects on patients and their families.

## Discussion

Oral cancer is emerging as one of the most challenging health problems among physicians due to its recurrence rate and the illicit knowledge and ill effects of tobacco products used despite knowing their adverse effects [6]. Siegel et al. (2024) stated that the estimated number of new cancer cases affecting the oral cavity and pharynx among the US population in 2024 was approximately 41,510 cases (4%) [7]. The World Health Organization (WHO) said that approximately 1 in 10 Indians will develop cancer during their lifetime and 1 in 15 will die of oral cancer. In India, oral cancer ranks as the sixth most common cancer, with approximately 120,000 cases (92,000 males and 28,000 females) reported in 2020 [8]. We anticipate that in 2020, the number of male tobacco-related cancer cases will be approximately 225,241, while the number of female tobacco-related cancer cases will rise to 93,563 [8]. Karnataka, Kerala, and Tamil Nadu reported the most number of oral cancer cases (97.42%), Telangana and Andhra Pradesh reported a relatively small proportion of cases (1.84%), and other Indian states accounted for 2664 records (0.74%) in 2024 [8].

Risk factors for oral cancer

The risk factors for oral cancer are usually multifactorial. The nitrosamines released from smokeless and smoking tobacco promote oral cancer formation. The practice of smoking in the form of a bidi or cigarette or chewing tobacco in the form of hans or gutkha, along with betel nut or betel quid, is still being practiced among the South Asian population [9]. Porphyromonas gingivalis might play a significant role in the growth of oral squamous cell carcinoma, possibly playing a part in three separate stages: the growth of cancerous cells, the change of cancerous cells from epithelial to mesenchymal, and the invasion of the tumor [9]. Other viruses linked to oral cancer include HPV-16 (approximately 98%), HPV-18, HSV-1 in conjunction with HPV as a cofactor, Epstein-Barr virus, and cytomegalovirus. Both smokeless and smoking or chewing forms of tobacco products cause oral cancer [9].

Areca nut raises the level of IL-6 and helps epithelial-mesenchymal interaction in oral keratinocytes, which leads to cancer [10]. The use of areca nut promotes oral submucous fibrosis, a predisposing, potentially malignant disorder that can lead to oral cancer. Along with the smoking or smokeless forms of tobacco, alcohol consumption also favors the formation of oral cancer as it favors the dryness of the oral mucosa, thereby preventing the clearance of harmful tobacco products and enhancing their retention in the oral mucosa. Researchers have linked an increased risk of oral cancer to age-related tooth wear, constant trauma on the buccal mucosa or lateral border of the tongue, constant trauma from dentures that are too short or too sharp, and the rise in quack dentists' use of wired treatment partial dentures made of self-cure acrylic resin. The Plummer-Vinson syndrome, a condition associated with iron deficiency anemia, increases oral cancer risk by forming esophageal strictures with a cricoid web. People who have dyskeratosis congenita, xeroderma pigmentosum, scleroderma, or Fanonis anemia are more likely to get oral cancer due to their genetic link to those conditions. Immunosuppressions such as HIV and uncontrolled diabetes mellitus are also likely to promote cancer progression. Older age is a risk factor associated with a poor prognosis for patients with squamous cell carcinoma of the oral cavity [10]. Oral cancer affects any area of the oral cavity, and its indications and symptoms vary depending on the anatomical location within the mouth. Oral squamous cell carcinoma grows in a number of ways, such as dividing cells more quickly, being able to invade and spread, avoiding immune system detection, stopping cells from dying naturally, encouraging blood vessel growth, and helping cancer stem cells [10].

The anterior tonsillar medially bounds the small, triangular-shaped retromolar area, which covers the anterior ramus of the mandible. It is located behind the third molar tooth. Clinically, cancer manifests as an ulcer, a growth, or an ulceroproliferative growth. The surface of this ulceroproliferative growth may also be whitish if there is food debris on it or if candidal microbial colonies are on top of it. This can happen if the ulcer is sloughing or burrowing [9,10]. The various warning signs of oral cancer include non-healing ulcers or proliferative growth. The warning signs of oral cancer include sudden mobility and even exfoliation of the teeth, spontaneous bleeding from the gums, sudden drifting of the teeth, sore throats, sudden deformation of the face resulting in facial asymmetry due to swelling from the underlying infection, dysphagia, and even the inability to swallow liquid food if cancer occurs in the pharynx [11].

Carcinoma of the cheek

Clinically, patients with carcinoma of the cheek typically complain of tooth mobility, mouth soreness while eating spicy foods, or extraoral swelling mimicking a space infection such as cellulitis or a dentoalveolar abscess. This type of cancer in the cheek can infect the fascial muscle planes below, resulting in a burrowing ulcer or an extraoral sinus opening on the external skin of the cheeks. When someone has had long-term cheek cancer, they typically have an ipsilaterally enlarged submandibular lymph node. This lymph node is usually stony-hard and fixed because the cancerous cells have spread beyond the capsular area of the lymph node and into the nearby muscle or facial planes [11-13]. We tabulated the reported cases of oral cancer with their chief complaint, clinical findings, assessment of TNM staging, prognosis, or outcome (Table 2).

**Table 2:** Reported cases of oral cancer.

Patient Age	Gender	Chief Complaint	Case History	Clinical Findings	Pathological Investigations Advised	TNM Staging Assessment	Treatment Plan	Prognosis/Treatment Outcome
57-year-old	Male	Sudden change and hoarseness in voice for the past one month	Bidi smoker: 3 bidis per day for 17 years	Ulceroproliferative growth involving the right retromolar region, gingiva, and posterior mandibular alveolar mucosa	Total count, differential count, bleeding time, clotting time	T3N1MX	Chemoradiotherapy [12]	Poor
52-year-old	Male	Soreness in the tongue for the past two weeks	Bidi smoker: 5 bidis per day for 14 years	Ulceroproliferative growth involving the right lateral border of the tongue	Total count, differential count, bleeding time, clotting time computed tomography (CT)	T2N0MX	Compartmental tongue surgery (CTS) followed by reconstruction with pedicled flaps such as pectoralis major myocutaneous flap, submental island flap, and latissimus dorsi pedicled flap. Surgery followed by post-adjuvant chemoradiotherapy [12-14]	Poor
62-year-old	Male	Soreness while eating food for the past two months	Bidi smoker for the past 18 years. He smokes about 12 bidis per day	Ulceroproliferative growth measuring about 1 x 1.5 cm on the right buccal mucosa in relation to the right mandibular second and third molar regions	Post-prandial blood glucose 247 mg/dL	T2N0MX	Surgery, chemoradiotherapy [15]	Poor
48-year-old	Male	Sudden drifting and spacing of his lower front teeth	Cigarette smoker 7 years. Smokes 7 cigarettes per day	A proliferative growth measuring about 1 x 1.5 cm on the gingiva, causing drifting caused by the pathological migration of the teeth of the underlying growth on the gingiva	Total count, differential count, bleeding time, clotting time, IOPA (Intraoral periapical radiograph), computed tomography (CT), bone scan/positron emission tomography (PET) to assess distant metastases	T3N1MX	Surgery, hemimandibulectomy, followed by reconstruction with titanium plates, radical segmental resection bilateral neck dissection [15-17]	Poor

Carcinoma of the posterolateral border of the tongue

Cancer affects the right posterolateral border of the tongue, causing an ulceroproliferative growth that is difficult to visualize without an endoscope. Patients with this type of cancer often complain of restricted tongue protrusion. Cancer that occurs near the tip of the tongue's ventral surface is typically presented as an irregularly shaped ulcer with an everted margin, necrotic slough, and an indurated base. Squamous cell carcinoma accounts for 90% of oral cavity cancers [14].

Bruschini et al. (2023) stated that in certain circumstances, a high function-sparing surgery with better postoperative functional outcomes is possible using a type IIIA glossectomy. From an oncological standpoint, it allows for drastic surgery without the need for postoperative radiation if there is no extracapsular dissemination, multiple nodal metastases, or involvement of the T-N tract in tongue cancers [15].

Carcinoma of the alveolus

Carcinoma affecting the mandible can result in sudden deformation of the face and associated paresthesia due to a cancer pathological fracture. Clinically, such affected patients present with a lump in the face or neck. Cancer in the maxillary posterior alveolar bone can cause erosion and inflammation of the ciliated columnar epithelial lining of the maxillary sinus. This can make you feel like you have chronic maxillary sinusitis, with symptoms like a stuffy nose, bad breath, and trouble breathing. If carcinoma occurs in the alveolar bone, it can break through the mandibular periosteal covering and invade the bone marrow structures. If the cancer spreads to the masseter muscle below, it can also cause trismus [18]. If it spreads, carcinoma of the alveolus can break through the bone's outermost periosteal covering. This can cause inflammation in the bone marrow's haversian canals, which increases osteoclastic activity. This, in turn, can lead to a pathological jaw fracture. The teeth in close proximity to cancer exhibit a "spiky appearance of the root tip" as a result of external root resorption, which occurs due to the cancer's rapid growth rate. This process results in tooth mobility and exfoliation [18,19].

Carcinoma on the gingiva

The gingiva suddenly enlarges in size within a short span of time and bleeds spontaneously. The gingival sulcular epithelium destroys the attached gingiva, leading to gingival recession. When such carcinoma affects the gingiva, there is a characteristic “floating teeth” appearance, which can mimic chronic periodontitis and chronic periodontal abscess. Shingaki et al. (2020) stated that the state of the surgical margins had a significant impact on how patients with mandibular gingival cancers fared [17].

Bharanidharan et al.(2015) stated that while gingival carcinomas can occur in dentate regions as well, they typically originate in edentulous locations. Most individuals concur that mandibular gingiva carcinomas are more prevalent than maxillary gingiva carcinomas, with 60% of the former occurring posterior to the premolars [18].

Moratin et al. (2020) suggested that tumors of the mandibular anterior region often have high rates of metastasis and local recurrence. Treatment for these patients usually involves bilateral neck dissection and radical segmental resection [19].

## Conclusions

Oral cancer represents a significant health concern globally due to its potential for serious complications and high mortality rates, particularly when diagnosed at advanced stages. A thorough history and a good visual examination can help the clinician diagnose this terrible cancer early and prevent mortality. The term "prevention is better than cure" always applies in the case of oral cancer. Increased awareness, public campaigning programs, and mass media communication are all critical to preventing oral cancer. Early detection, through regular screenings and awareness of risk factors, is key to improving survival rates and reducing the impact of this disease on individuals and communities. Efforts to promote tobacco cessation, limit alcohol consumption, encourage a healthy diet, and vaccinate against HPV can also contribute to the prevention of oral cancer. Moreover, ongoing research into innovative diagnostic tools, treatment modalities, and supportive care strategies is essential for further advancements in the management of this challenging condition.
